# Interpersonal Violence Victimization Among High School Students — Youth Risk Behavior Survey, United States, 2019

**DOI:** 10.15585/mmwr.su6901a4

**Published:** 2020-08-21

**Authors:** Kathleen C. Basile, Heather B. Clayton, Sarah DeGue, John W. Gilford, Kevin J. Vagi, Nicolas A. Suarez, Marissa L. Zwald, Richard Lowry

**Affiliations:** ^1^Division of Violence Prevention, National Center for Injury Prevention and Control, CDC; ^2^Division of Adolescent and School Health, National Center for HIV/AIDS, Viral Hepatitis, STD, and TB Prevention, CDC; ^3^Office of the Director, National Center for HIV/AIDS, Viral Hepatitis, STD, and TB Prevention, CDC

## Abstract

Adolescent interpersonal violence victimization is an adverse childhood experience and a serious public health problem for youths, their families, and communities. Violence victimization includes dating violence, sexual violence, and bullying. Youth Risk Behavior Survey data for 2019 were used to examine physical and sexual dating violence; sexual violence by anyone; and bullying victimization, whether on school property or electronic, of U.S. high school students by sex, race/ethnicity, and sexual identity. In addition, this report explores frequency of dating violence and frequency of sexual violence among students who reported these forms of victimization and presents composites of dating violence and bullying. Findings reveal that 8.2% of students reported physical dating violence; 8.2% reported sexual dating violence; 10.8% reported sexual violence by anyone, of which 50% of cases were by a perpetrator other than a dating partner; 19.5% reported bullying on school property; and 15.7% reported electronic bullying victimization during the previous 12 months. Approximately one in eight students reported any dating violence, and one in four reported any bullying victimization. Female students; lesbian, gay, and bisexual students; and students not sure of their sexual identity reported the highest prevalence estimates across all five violence victimization types, any and both forms of dating violence, and any bullying victimization. Non-Hispanic white students reported the highest prevalence of bullying victimization. Among students experiencing physical or sexual dating violence or sexual violence by anyone, the most common frequency reported was one time during the previous year; higher frequency was more prevalent among male students compared with female students. These findings provide a contextual understanding of the prevalence of interpersonal violence of U.S. high school students, highlighting those with highest prevalence. Findings can be used by public health professionals to guide prevention efforts with youths in schools and communities.

## Introduction

Interpersonal violence, or aggression perpetrated by another person, including dating violence, sexual violence, and bullying, is a serious problem for students, schools, and communities. Violence can reoccur across the lifespan and is associated with multiple health effects and negative health behaviors (e.g., risky sexual behaviors, substance misuse, and physical health symptoms) (*1*). Victimization often begins during adolescence and can be viewed as an adverse childhood experience (ACE). For example, nationally representative data from adults during 2015 indicate that 43.2% of females and 51.3% of males who had been raped were first raped before age 18 years (*2*). Prevalence studies of adolescents confirm this finding. For example, a survey of students in grades 7–12 found that 56% of females and 48% of males reported some form of sexual violence victimization by a peer (e.g., unwelcome comments, touching, or being forced to do something sexual) during the 2010–11 school year (*3*). Approximately 20% of adolescents reported physical dating violence and 9% reported sexual dating violence (*4*). These studies indicate that sexual violence during adolescence occurs inside and outside of the dating context. In addition, 20% of students in grades 6–12 reported bullying victimization during the 2017 school year (*5*).

Scientific literature indicates that certain groups (e.g., females, racial/ethnic minorities, and sexual minority youths) disproportionately experience interpersonal violence during adolescence (*1*). For instance, in a sample of northeastern 10th-grade students, sexual minority youths reported more bullying, sexual violence, and dating violence victimization than heterosexual youths, with sexual minority females reporting particularly high levels (91% of sexual minority females and 79% of sexual minority males reported at least one form of victimization) (*6*). Furthermore, in a study of sexual violence victimization of college students, females had higher odds of victimization than did males, and non-Hispanic black (black) students and students of other races/ethnicities had higher odds of victimization than did non-Hispanic white (white) students; moreover, these racial differences were greater for males. For females, Hispanics had lower odds of sexual violence victimization than whites, and for males, no substantial differences existed between Hispanics and whites (*7*). Understanding these disparities in the experience of violence victimization is crucial for identifying those at highest risk and for guiding prevention efforts. Contextual factors also are valuable in describing victimization (e.g., frequency of victimization or co-occurrences of violence subtypes). These factors increase understanding of these violence types and further contextualize prevalence estimates. For example, in a report using 2013 data, approximately 21% of female and 10% of male high school students who reported dating in the previous year experienced sexual or physical dating violence, and 6% of females and 3% of males experienced both physical and sexual dating violence (*8*).

This report presents 2019 prevalence estimates for dating violence, sexual violence, and bullying victimization of U.S. high school students by sex, race/ethnicity, and sexual identity, and includes frequency of dating violence and sexual violence victimization by demographic characteristics. Combined prevalence of different forms of dating violence and bullying also is presented to provide the most current estimates of each violence type. These findings can guide prevention efforts in addressing adolescent interpersonal violence at different levels of the social ecology (i.e., individual, relationship, and community or societal levels).

## Methods

### Data Source

This report includes data from CDC’s 2019 Youth Risk Behavior Survey (YRBS), a cross-sectional, school-based survey conducted biennially since 1991. Each survey year, CDC collects data from a nationally representative sample of public and private school students in grades 9–12 in the 50 U.S. states and the District of Columbia (N = 13,677). Additional information about YRBS sampling, data collection, response rates, and processing is available in the overview report of this supplement (*9*). The prevalence estimates for all violence questions for the overall study population and by sex, race/ethnicity, grade, and sexual orientation are available at https://nccd.cdc.gov/youthonline/App/Default.aspx. The full YRBS questionnaire is available at https://www.cdc.gov/healthyyouth/data/yrbs/pdf/2019/2019_YRBS-National-HS-Questionnaire.pdf.

### Measures

This analysis included five standard measures of violence victimization and three composite variables created from those standard measures. The standard measures included 1) having experienced physical dating violence, 2) having experienced sexual dating violence, 3) having experienced sexual violence by anyone, 4) having been bullied on school property, and 5) having been bullied electronically (Table 1). For each of these five standard measures, dichotomous categories were created: ≥1 time versus 0 times for all sexual violence and dating violence measures and “yes” versus “no” for both bullying victimization measures. The manner in which the data were collected (see Limitations) means that approximately 25% of respondents were missing data for sexual violence victimization by anyone (3,439) out of a sample of 13,677 students. The denominators for dating violence victimization measures are students who reported dating during the 12 months before the survey (66.1% [n = 8,703 students] for physical dating violence victimization and 66.2% [n = 6,847 students] for sexual dating violence victimization), whereas the denominator for the sexual violence by anyone and bullying victimization measures are the full sample of students for which data were available. Three of these standard measures included levels of victimization frequency. For each of three measures (i.e., physical dating violence, sexual dating violence, and sexual violence by anyone), frequencies were collapsed into three levels: 1 time, 2 or 3 times, or ≥4 times.

**TABLE 1:** Violence victimization measures — Youth Risk Behavior Survey, United States, 2019

Violence victimization	Questionnaire item	Coding for analysis
Physical dating violence victimization	“During the past 12 months, how many times did **someone you were dating or going out with** physically hurt you on purpose? (Count such things as being hit, slammed into something, or injured with an object or weapon.)” [Question excludes students who did not date or go out with anyone during the previous 12 months.]	≥1 time versus 0 times; 1 time, 2 or 3 times, ≥4 times
Sexual dating violence victimization*	“During the past 12 months, how many times did **someone you were dating or going out with** force you to do sexual things that you did not want to do? (Count such things as kissing, touching, or being physically forced to have sexual intercourse.)” [Question excludes students who did not date or go out with anyone during the previous 12 months.]	≥1 time versus 0 times; 1 time, 2 or 3 times, ≥4 times
Sexual violence victimization by anyone^†^	“During the past 12 months, how many times did **anyone** force you to do sexual things that you did not want to do? (Count such things as kissing, touching, or being physically forced to have sexual intercourse.)”	≥1 time versus 0 times; 1 time, 2 or 3 times, ≥4 times
Bullied on school property	“During the past 12 months, have you ever been bullied on school property?”	Yes versus no
Electronically bullied	“During the past 12 months, have you ever been electronically bullied?”	Yes versus no

**Abbreviation:** YRBS = Youth Risk Behavior Survey.

* A total of 3,324 students had missing data for this variable, mostly attributed to the use of different versions of the YRBS questionnaire that did not include the sexual violence questions in certain selected schools.

^†^ A total of 3,439 students had data missing for this variable, mostly attributed to the use of different versions of the YRBS questionnaire that did not include the sexual violence questions in certain selected schools.

The two dating violence victimization measures were combined into composite measures: experienced any dating violence victimization and experienced both physical and sexual dating violence victimization. Because of the manner in which the data were collected, approximately 25% of respondents were missing data for sexual dating violence victimization (3,324 observations out of a sample of 13,677 students). When calculating the “any dating violence victimization” measure, responses missing data for either the sexual or the physical dating violence measure were removed from the analysis. Any “yes” responses to either the physical dating violence measure or the sexual dating violence measure were combined for the numerator, with all responses without missing data as the denominator. Similarly, to create the “both physical and sexual dating violence” measure, “yes” responses to both physical dating violence and sexual dating violence were required for the numerator, with all nonmissing responses in the denominator. A similar strategy was also used for creating a bullying victimization “any” measure. “Any bullying victimization” included any “yes” response to either experiencing bullying at school or experiencing electronic bullying, with all nonmissing responses in the denominator. The option of exploring “both bullying at school and electronic bullying” was not pursued. Use of personal electronic devices in the school setting is increasing; therefore, the amount of overlap between electronic bullying and bullying at school might be considerable and combining these items could result in an overestimate of their prevalence. Additional analysis examined overlap between the sexual dating violence measure and the sexual violence by anyone measure.

Three demographic characteristics were included in the analyses: student sex (male or female), race/ethnicity (white, black, Hispanic, or other), and sexual identity (heterosexual; lesbian, gay, or bisexual [LGB]; or not sure). Although students of multiple or other race/ethnicity are included in these analyses, data are not presented for this group because small sample sizes and unknown heterogeneity within this group resulted in limited interpretability.

### Analysis

Weighted prevalence estimates and corresponding 95% confidence intervals were determined for all violence victimization measures. Comparisons by demographic characteristics were conducted with the chi-square test (p<0.05). When differences among groups were demonstrated, additional *t*-tests were performed to determine pairwise differences between groups. Differences between prevalence estimates were considered statistically significant if the *t*-test p value was <0.05 for main effects (sex, race/ethnicity, or sexual identity).

## Results

Among the approximately two thirds of U.S. high school students who reported dating during the 12 months before the survey, 8.2% reported experiencing physical dating violence, and 8.2% experienced sexual dating violence (Table 2). Sexual violence victimization perpetrated by anyone during the 12 months before the survey was reported by 10.8% of students. When comparing the sexual dating violence measure with the sexual violence by anyone measure, half (50%) of the 10.8% of students who reported sexual violence by anyone were victimized only by someone other than a dating partner. Experiences of bullying victimization during the 12 months before the survey varied, with 15.7% of students reporting experiencing electronic bullying and 19.5% reporting bullying on school property. For all violence victimization measures, the prevalence varied by both sex and sexual identity, and variation by race/ethnicity was only observed for bullying victimization. Specifically, female students, LGB students, and students not sure of their sexual identity consistently had the highest prevalence across all five of the violence victimization indicators. In addition, compared with Hispanic or black students, white students had the highest prevalence of experiencing bullying victimization at school and electronic bullying. The prevalence of electronic bullying among Hispanic students was also significantly greater than the prevalence among black students.

**TABLE 2:** Percentage of high school students who experienced violence victimization,* by demographic characteristics and type of violence — Youth Risk Behavior Survey, United States, 2019

Characteristic	Experienced physical dating violence^†^		Experienced sexual dating violence^§^		Experienced sexual violence by anyone^¶^	
	% (95% CI)	p value**	% (95% CI)	p value**	% (95% CI)	p value**
**Total**	**8.2 (7.2–9.4)**	**NA**	**8.2 (7.4–9.1)**	**NA**	**10.8 (9.9–11.7)**	**NA**
**Sex**						
Female	9.3 (8.0–10.8)	0.01	12.6 (11.2–14.2)	<0.01	16.6 (15.1–18.2)	<0.01
Male	7.0^††^ (5.8–8.4)	NA	3.8^††^ (3.1–4.7)	NA	5.2^††^ (4.4–6.1)	NA
**Race/Ethnicity**						
White, non-Hispanic	7.5 (6.4–8.7)	0.43	8.1 (6.9–9.6)	0.11	10.2 (9.1–11.4)	0.23
Black, non-Hispanic	8.2 (6.1–10.8)	NA	6.2 (4.5–8.6)	NA	10.3 (8.0–13.1)	NA
Hispanic	8.9 (7.4–10.8)	NA	8.7 (6.9–10.8)	NA	12.2 (10.6–14.0)	NA
**Sexual identity**						
Heterosexual	7.2 (6.2–8.3)	0.01	6.7 (5.9–7.5)	<0.01	9.0 (8.2–9.9)	<0.01
Lesbian, gay, or bisexual	13.1^§§^ (10.5–16.1)	NA	16.4^§§^ (12.7–20.9)	NA	21.5^§§^ (18.2–25.2)	NA
Not sure	16.9^§§^ (11.1–24.9)	NA	15.0^§§^ (9.5–23.0)	NA	16.2^§§ ^(11.7–22.0)	NA
**Characteristic**	**Bullied on school property**		**Electronically bullied**		—	—
**Total**	**19.5 (18.2–20.9)**	NA	**15.7 (14.6–16.9)**	NA	—	—
**Sex**						
Female	23.6 (21.8–25.5)	<0.01	20.4 (18.9–22.0)	<0.01	—	—
Male	15.4^§§^ (14.0–16.9)	NA	10.9^††^ (9.6–12.4)	NA	—	—
**Race/Ethnicity**						
White, non-Hispanic	23.1 (21.4–24.8)	<0.01	18.6 (17.1–20.2)	<0.01	—	—
Black, non-Hispanic	15.1^¶¶^ (13.1–17.4)	NA	8.6^¶¶ ^(7.4–10.0)	NA	—	—
Hispanic	14.8^¶¶^ (12.8–17.1)	NA	12.7^¶¶,^*** (11.1–14.5)	NA	—	—
**Sexual identity**						
Heterosexual	17.1 (15.7–18.7)	<0.01	14.1 (12.9–15.4)	<0.01	—	—
Lesbian, gay, or bisexual	32.0^§§^ (29.5–34.6)	NA	26.6^§§^ (23.3–30.2)	NA	—	—
Not sure	26.9^§§^ (22.2–32.2)	NA	19.4^§§,†††^ (15.5–24.0)	NA	—	—

**Abbreviations:** CI = confidence interval; NA = not applicable; YRBS = Youth Risk Behavior Survey.

* During the 12 months before the survey.

^†^ Being physically hurt on purpose (counting such things as being hit, slammed into something, or injured with an object or weapon) by someone they were dating or going out with, ≥1 time, among the 66.1% (n = 8,703) of students nationwide who dated or went out with someone during the 12 months before the survey.

^§^ Being forced to do “sexual things” (counting such things as kissing, touching, or being physically forced to have sexual intercourse) they did not want to do by someone they were dating or going out with, ≥1 time, among the 66.2% (n = 6,847) of students nationwide who dated or went out with someone during the 12 months before the survey. Of 13,677 students, this variable was missing for 3,324, mostly attributed to the use of different versions of the YRBS questionnaire that did not include the sexual violence questions in certain selected schools. This resulted in complete data for 10,353 students, of which 66.2% (6,847) reported dating in the 12 months before the survey.

^¶^ Being forced to do “sexual things” (counting such things as kissing, touching, or being physically forced to have sexual intercourse) they did not want to do by anyone, ≥1 time, during the 12 months before the survey. Data were missing for 3,439 students for this variable, mostly attributed to the use of different versions of the YRBS questionnaire that did not include the sexual violence questions in certain selected schools.

** Chi-square test (p<0.05).

^††^ Significantly different from female students, based on *t*-test (p<0.05).

^§§^ Significantly different from heterosexual students, based on *t*-test (p<0.05).

^¶¶^ Significantly different from white students, based on *t*-test (p<0.05).

*** Significantly different from black students, based on *t*-test (p<0.05).

^†††^ Significantly different from lesbian, gay, or bisexual students, based on *t*-test (p<0.05).

Among students who experienced physical dating violence, sexual dating violence, or sexual violence by anyone during the previous year, the most common frequency reported was 1 time for each (Figure). The pattern of frequency for violence victimization differed by type of victimization. The distribution of frequency for physical dating violence victimization was U-shaped, with the highest levels of frequency at 1 time and ≥4 times, whereas for both sexual dating violence victimization and sexual violence victimization by anyone, the most common frequency was 1 time, with a decreasing prevalence as the frequency increased.

**FIGURE:**
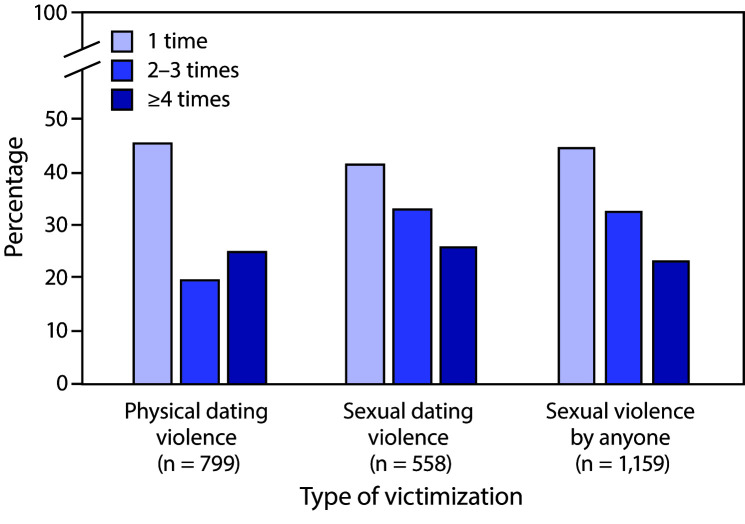
Percentage of high school students who experienced violence, by type of victimization (physical dating violence, sexual dating violence, or sexual violence by anyone) and by number of times during the previous year — Youth Risk Behavior Survey, United States, 2019

The frequency of physical and sexual dating violence varied significantly by sex (Table 3). Specifically, the prevalence of physical dating violence was significantly greater at higher frequency levels (≥4 times) among male students compared with female students (41.6% versus 21.6%, respectively). This frequency distribution pattern was similar for sexual dating violence. The prevalence at the higher end of frequency for sexual dating violence was significantly greater for male students compared with female students (41.0% versus 20.8%, respectively). Higher frequency (≥4 times) was also reported for sexual violence by anyone for male students compared with female students (33.9% versus 18.6%, respectively). No significant differences existed by race/ethnicity in frequency of physical and sexual dating violence or sexual violence by anyone. These analyses could not include sexual identity because of limited data (i.e., group counts <30).

**TABLE 3:** Frequency of types of violence victimization,* by demographic characteristics among high school students reporting experiencing specific types of violence — Youth Risk Behavior Survey, United States, 2019

Type of violence victimization	Sex			Race/Ethnicity			
	Male % (95% CI)	Female % (95% CI)	p value^†^	White, non-Hispanic % (95% CI)	Black, non-Hispanic % (95% CI)	Hispanic % (95% CI)	p value^†^
**Experienced physical dating violence^§^**	NA	NA	<0.01	NA	NA	NA	0.21
1 time	38.0 (32.2–44.2)	51.7 (44.2–59.2)	NA	47.9 (39.7–56.2)	47.5 (37.6–57.7)	40.9 (31.3–51.3)	NA
2 or 3 times	20.4 (14.2–28.4)	26.7 (21.6–32.5)	NA	25.5 (18.7–33.8)	16.7 (10.3–25.9)	27.0 (19.5–36.0)	NA
≥4 times	41.6 (34.6–48.9)	21.6 (16.9–27.1)	NA	26.6 (20.1–34.3)	35.8 (25.0–48.2)	32.1 (24.9–40.3)	NA
**Experienced sexual dating violence^¶^**	NA	NA	0.05	NA	NA	NA	0.39
1 time	33.3 (23.8–44.4)	44.0 (36.5–51.8)	NA	42.2 (33.7–51.2)	29.0 (15.5–47.6)	45.0 (33.3–57.3)	NA
2 or 3 times	25.7 (16.8–37.2)	35.2 (28.4–42.6)	NA	32.3 (25.8–39.4)	38.6 (23.8–56.0)	33.3 (22.3–46.5)	NA
≥4 times	41.0 (28.0–55.3)	20.8 (15.3–27.6)	NA	25.5 (18.8–33.7)	32.4 (15.4–55.7)	21.6 (14.4–31.2)	NA
Experienced sexual violence by anyone**	NA	NA	0.01	NA	NA	NA	0.36
1 time	36.6 (28.7–45.4)	47.3 (42.8–52.0)	NA	47.6 (41.2–54.1)	39.7 (30.0–50.2)	44.0 (36.6–51.6)	NA
2 or 3 times	29.5 (21.8–38.6)	34.1 (29.9–38.5)	NA	31.2 (26.3–36.6)	34.9 (27.1–43.7)	34.5 (28.1–41.6)	NA
≥4 times	33.9 (25.3–43.8)	18.6 (15.2–22.5)	NA	21.2 (16.1–27.4)	25.4 (17.1–36.0)	21.5 (15.7–28.7)	NA

**Abbreviations: **CI = confidence interval; NA = not applicable; YRBS = Youth Risk Behavior Survey.

* During the 12 months before the survey.

^†^ Chi-square test (p<0.05).

^§^ Being physically hurt on purpose (counting such things as being hit, slammed into something, or injured with an object or weapon) by someone they were dating or going out with, ≥1 time, among the 66.1% (n = 8,703) of students nationwide who dated or went out with someone during the 12 months before the survey.

^¶^ Being forced to do “sexual things” (counting such things as kissing, touching, or being physically forced to have sexual intercourse) they did not want to do by someone they were dating or going out with, ≥1time, among the 66.2% (n = 6,847) of students nationwide who dated or went out with someone during the 12 months before the survey. Of 13,677 students, this variable was missing for 3,324, mostly attributed to the use of different versions of the YRBS questionnaire that did not include the sexual violence questions in certain selected schools. This resulted in complete data for 10,353 students, of which 66.2% (6,847) reported dating in the 12 months before the survey.

** Being forced to do “sexual things” (counting such things as kissing, touching, or being physically forced to have sexual intercourse) they did not want to do by anyone during the 12 months before the survey. These data were missing for 3,439 students for this variable, mostly attributed to the use of different versions of the YRBS questionnaire that did not include the sexual violence questions in certain selected schools.

Overall, 12.2% of students experienced any type of dating violence victimization, and 3.0% experienced both types (Table 4). Both dating violence composite measures varied substantially by sex and sexual identity but not by race/ethnicity. The prevalence of the dating violence composite variables was significantly greater for female students compared with male students (16.4% versus 8.2% for any dating violence type; 3.8% versus 2.1% for both dating violence types). Students who did not identify as heterosexual had substantially greater prevalence of both dating violence composites. For any type of dating violence, the prevalence was 22.3% for LGB students and 18.7% for students who were not sure of their sexual identity versus 10.5% for heterosexual students. For both types of dating violence, the prevalence was 5.8% for LGB students and 9.4% for students not sure of their sexual identity versus 2.4% for heterosexual students.

**TABLE 4:** Percentage of high school students who experienced any dating violence or both physical and sexual dating violence* and any form of bullying victimization,^†^ by demographic characteristics — Youth Risk Behavior Survey, United States, 2019

Characteristic	Dating violence composite variables				Bullying victimization composite	
	Experienced any dating violence^§^		Experienced both physical and sexual dating violence^¶^		Experienced any bullying**	
	% (95% CI)	p value^††^	% (95% CI)	p value^††^	% (95% CI)	p value^††^
**Total**	**12.2 (11.3–13.3)**	**NA**	**3.0 (2.5–3.7)**	NA	**24.8 (23.4–26.3)**	NA
**Sex**						
Female	16.4 (14.7–18.2)	<0.01	3.8 (3.0–5.0)	0.01	30.2 (28.4–32.1)	<0.01
Male	8.2^§§^ (7.1–9.4)	NA	2.1^§§^ (1.6–2.9)	NA	19.2^§§^ (17.6–20.9)	NA
**Race/Ethnicity**						
White, non-Hispanic	12.1 (10.8–13.5)	0.42	2.8 (2.2–3.5)	0.51	28.8 (26.9–30.7)	<0.01
Black, non-Hispanic	10.6 (7.9–14.1)	NA	3.0 (1.7–5.2)	NA	18.0^¶¶^ (15.7–20.6)	NA
Hispanic	12.7 (11.1–14.6)	NA	3.3 (2.1–5.1)	NA	19.2^¶¶^ (17.4–21.1)	NA
**Sexual identity**						
Heterosexual	10.5 (9.5–11.6)	<0.01	2.4 (2.0–2.9)	0.01	22.2 (20.6–23.8)	<0.01
Lesbian, gay, or bisexual	22.3*** (17.9–27.5)	NA	5.8*** (3.9–8.4)	NA	39.5*** (36.6–42.5)	NA
Not sure	18.7*** (13.2–26.0)	NA	9.4*** (5.0–16.9)	NA	32.7***^,†††^ (27.6–38.3)	NA

**Abbreviations**: CI = confidence interval; NA = not applicable.

* During the 12 months before the survey, among students who dated or went out with someone during the 12 months before the survey.

^†^ During the 12 months before the survey.

^§^ Combined any “yes” responses to physical dating violence and sexual dating violence. Because of the manner in which this variable was calculated, missing values in both the physical dating violence and sexual dating violence measures resulted in 3,355 missing values in the “experienced any dating violence” composite measure.

^¶^ Combined where responses to both physical dating violence and sexual dating violence were “yes.” Because of the manner in which this variable was calculated, the missing values in both the physical dating violence and sexual dating violence measures resulted in 3,355 missing observations in the “experienced both physical and sexual dating violence” composite measure.

** Combined any “yes” responses to bullied at school and electronic bullying.

^††^ Chi-square test (p<0.05).

^§§^ Significantly different from female students, based on *t*-test (p<0.05).

^¶¶^ Significantly different from white, non-Hispanic students, based on *t*-test (p<0.05).

*** Significantly different from heterosexual students, based on *t*-test (p<0.05).

^†††^ Significantly different from lesbian, gay, or bisexual students, based on *t*-test (p<0.05).

The prevalence of experiencing any type of bullying victimization was 24.8% (Table 4), and prevalence varied significantly by sex, race/ethnicity, and sexual identity. The prevalence of experiencing any bullying victimization was significantly greater for female students compared with male students (30.2% versus 19.2%, respectively) and significantly greater for white (28.8%) compared with black (18.0%) or Hispanic (19.2%) students. Both LGB students (39.5%) and students not sure of their sexual identity (32.7%) had significantly higher prevalence of any bullying compared with heterosexual students (22.2%), with LGB students reporting greater prevalence than students not sure of their sexual identity.

## Discussion

This report describes the 2019 prevalence and frequency of different forms of interpersonal violence victimization experienced by U.S. high school students. Similar to findings from previous YRBSs (https://www.cdc.gov/violenceprevention/pdf/2012FindingsonSVinYouth-508.pdf), physical dating violence, sexual dating violence, sexual violence by anyone, bullying on school property, and electronic bullying victimization are adverse childhood experiences (ACEs) that are occurring at high rates. Examining their prevalence individually and in combination by key demographic characteristics provides an overall observation and contextual understanding of interpersonal violence experienced by U.S. high school students and helps identify disparities in health and safety among U.S. youths, which can guide prevention efforts.

All five types of victimization, including any or both forms of dating violence and any form of bullying, were more common among female and sexual minority students, highlighting their more frequent victimization. These findings are consistent with previous studies that reported disparities in interpersonal violence victimization, particularly dating violence and sexual violence, by sex and sexual identity (*6**,**7*). Although findings did not reveal substantially greater prevalence for racial/ethnic minority youths for the forms of violence examined, research has consistently shown that racial/ethnic minority youths are at greater risk for homicides and other community violence victimization (https://www.cdc.gov/violenceprevention/pub/technical-packages.html). Disparities in health and risk for violence have been linked to sexism, homophobia, and structural disadvantage (*10*).

Half of students who reported sexual violence victimization by anyone did not report sexual violence by a dating partner, indicating that students who experience sexual violence are often victimized by someone other than a dating partner. This finding is consistent with previous research (*3*) documenting that sexual violence happening in school during adolescence is frequently perpetrated by peers and not necessarily by dating partners. Indeed, perpetrators of sexual violence during youth can be acquaintances, family members, persons in a position of authority, and strangers, in addition to dating partners (https://www.cdc.gov/violenceprevention/pdf/2012FindingsonSVinYouth-508.pdf). This indicates that efforts might need to be focused on preventing sexual violence both inside and outside the context of dating relationships to be most helpful.

Males who experienced dating violence or sexual violence reported high frequencies of victimization (≥4 times during the previous year) substantially more often than did females. That is, although male students do not report higher prevalence of victimization than do female students, when they do report it, they report experiencing it at a higher frequency. Previous research has documented that, among youths at high risk (i.e., previously exposed to violence in the home or community), adolescent males reported higher frequency of victimization than did females for sexual dating violence (*11*). However, male adolescents might also be more likely to disclose dating violence and sexual violence when the victimization has happened more than once.

In this study, bullying victimization was the only type of violence victimization examined for which racial/ethnic differences existed, with substantially higher prevalence occurring among white students compared with black or Hispanic students. This result for bullying is supported in part by previous research (*12*). In addition, Hispanic students reported substantially higher prevalence of electronic bullying victimization compared with black students. Other research has indicated that black students might underreport bullying victimization when presented with a definition-based measure of bullying that includes a form of the word “bully,” as is used in YRBS, as opposed to behaviorally specific measures that describe the victimization behaviors but do not use the word “bully” (*13*). The measurement of bullying in this study might have differentially affected reporting across racial/ethnic groups.

Overall, these findings highlight the importance of early engagement in effective, evidence-based efforts for preventing violence victimization and perpetration before they begin or stopping them from continuing. Findings from this study also demonstrate substantial differences in exposure to these types of violence by sex, race/ethnicity, and sexual identity, highlighting the need for prevention efforts that address the unique needs of these groups. To help communities focus their prevention efforts on what works and to address risk and protective factors for violence and other ACEs across the social ecology, CDC developed a series of technical packages that identify key violence prevention strategies and approaches on the basis of the best available research evidence. (CDC’s technical packages for violence prevention are available at https://www.cdc.gov/violenceprevention/pub/technical-packages.html.) This series includes packages focused on sexual violence, intimate partner violence (including dating violence), and youth violence (including bullying). *Preventing Adverse Childhood Experiences (ACEs): Leveraging the Best Available Evidence* compiles evidence focused on ACEs from across the technical packages (https://www.cdc.gov/violenceprevention/pdf/preventingACES.pdf).

Multiple evidence-based interpersonal violence prevention approaches are directly related to the findings in this study. For example, social-emotional learning programs that support development of skills for communication, emotion regulation, empathy, and respect and that target risk factors for interpersonal violence (e.g., impulsivity or drug use) have been reported to decrease adolescent sexual violence perpetration and homophobic name-calling, with indirect effects on peer bullying, cyberbullying, and sexual harassment perpetration when mediated by delinquency (*14*,*15*). By addressing shared risk and protective factors across types of violence, social-emotional learning programs can build the skills youths need for engaging in healthy relationships with family, peers, dating partners, and others, thus preventing multiple forms of adolescent interpersonal violence and long-term consequences into adulthood. In addition, bystander programs teach youths how to safely act when they see behaviors that increase risk for violence and change social norms within their peer groups. Although originally conceptualized as a means of challenging heterosexist attitudes to prevent sexual and dating violence (*16*), such programs might also prevent other forms of adolescent violence, including bullying and violence targeting sexual, gender, and racial minorities by focusing the training on recognizing and challenging these specific harmful attitudes and behaviors (*17*,*18*).

Modifying the social and physical environment in schools and neighborhoods might improve safety and reduce risk for violence for more of the population than individual- or relationship-level approaches alone. For example, one school-based prevention approach that includes a building-level intervention (e.g., addressing physical areas in the school identified by students as less safe) has been reported to reduce sexual violence victimization and perpetration by peers and dating partners (*19*). In addition, the development of safe and supportive environments in schools that promote protective factors (e.g., school connectedness and professional development regarding lesbian, gay, bisexual, and transgender [LGBT] youths) can help create accepting school environments for LGBT youths and reduce the risk for bullying and other violence (*20*). Results from this report indicate that LGB youths, specifically, are at a disproportionately higher risk for interpersonal violence victimization compared with heterosexual youths. As of 2019, gender identity has not been assessed by the YRBS nationwide. However, during 2017, gender identity was assessed in YRBSs conducted in 10 states and nine large urban school districts; these data show that transgender students consistently report greater prevalence of violence victimization than their cisgender peers (*21*). Promotion of gay-straight alliances and support of LGBT students can help provide these youths with an accepting school environment, which might also reduce the risk for school-based violence against these youths (*22*). (Information about CDC’s current school health programs is available at https://www.cdc.gov/healthyyouth/fundedprograms/1807/resources.htm.)

CDC is engaged in ongoing research and programmatic activities for expanding the research evidence and adding to the knowledge base of effective primary prevention programs, policies, and practices available to communities for preventing interpersonal violence among youths. For example, CDC’s *Dating Matters: Strategies to Promote Healthy Teen Relationships* is a comprehensive adolescent dating violence prevention model. *Dating Matters* includes multiple integrated prevention strategies that address risk factors for youths and their families, schools, and neighborhoods with demonstrated effects on adolescent dating violence, bullying, and peer violence in middle school. (Additional information about *Dating Matters* is available at https://www.cdc.gov/violenceprevention/intimatepartnerviolence/datingmatters/index.html.) 

In addition, since 2001, CDC has provided funding for primary prevention of sexual violence through the Rape Prevention and Education Program to state health departments in all 50 states, the District of Columbia, and four U.S. territories. Funded organizations implement initiatives that address youths in their communities, including community- and societal-level approaches (e.g., improving education and leadership opportunities for girls). (Additional information about the Rape Prevention and Education Program is available at https://www.cdc.gov/violenceprevention/sexualviolence/rpe/index.html.) CDC also sponsors youth violence prevention research through its National Centers of Excellence in Youth Violence Prevention. Their goal is to build the scientific infrastructure and community partnerships necessary for stimulating new youth violence prevention research and practice across the country, including a focus on the impact of structural factors (e.g., housing, education, or systemic discrimination) that limit access to positive social determinants of health.

Prevention of interpersonal violence among adolescents might be most successful when a comprehensive strategy is used that addresses these ACEs at multiple levels of the social ecology simultaneously and recognizes that these different forms of victimization can be co-occurring (*1*). The findings reported here also highlight the importance of acknowledging the disproportionate prevalence of these forms of victimization on certain youths (i.e., females and sexual minorities) and addressing these disparities in prevention efforts.

## Limitations

General limitations for the YRBS are available in the overview report of this supplement (*9*).The findings in this report are subject to at least five additional limitations. First, substantial overlap likely existed in the measures that examined experiences of sexual violence victimization (i.e., sexual dating violence victimization and sexual violence victimization by anyone), and among the bullying victimization measures (i.e., electronic bullying and bullied at school). For these reasons, composites for the sexual violence measures and a “both” composite for bullying (i.e., experienced both electronic bullying and bullying at school) were not created. Second, because of the breadth of topics included in the YRBS, violence victimization subtype measures included in the YRBS tend to be broad in nature and, in this study, were assessed by single items. More specific and detailed measures of violence victimization would allow for a comprehensive analysis of the prevalence and overlap between different forms of interpersonal violence victimization. Third, the YRBS bullying items include the word “bullied,” which might have decreased disclosure (*13*). Fourth, the interpersonal violence victimization types that could be included in this study (i.e., dating violence, sexual violence, and bullying), as a whole, do not reflect the breadth of interpersonal violence victimization experienced by youths (i.e., other forms of youth violence experienced in the community) and might partially explain why few racial/ethnic differences were found. Finally, the sexual violence measures (and composite measures that were created with the sexual violence measures) in this report had a relatively large amount of missing data (approximately 3,400 observations) in 2019. Most of this missing data can be attributed to the use of different versions of the YRBS questionnaire that did not include the sexual violence questions in certain selected schools. Consequently, not all students in the national sample were given the opportunity to answer the sexual violence questions and were counted as missing. When constructing the composite measures for any dating violence, and both physical and sexual dating violence victimization, the analytic sample was restricted to students who had complete data for both physical and sexual dating violence victimization, which reduced the potential for biased estimates. 

## Future Directions

To increase understanding of the differential experiences of adolescent interpersonal violence victimization, future research that focuses in more detail on the demographic groups highlighted in this study can be beneficial. For example, on the basis of these findings, additional research to better understand the characteristics and consequences of these forms of interpersonal violence on sexual minority youths is warranted. Research exploring sex differences in the frequency of victimization across additional types of violence can add to the findings reported here. Future studies that include more detailed measures of dating violence, sexual violence, and bullying for capturing and isolating understudied subtypes of these forms of violence (e.g., psychological dating violence, nonconsensual sexting, or relational bullying) would increase knowledge of the full prevalence of these forms of violence among youths. Finally, studies that examine the co-occurrence and cumulative impact of different forms of violence victimization during adolescence and into adulthood can guide more comprehensive prevention efforts.

## Conclusion

Interpersonal violence victimization experiences of high school students are a form of ACEs and represent a substantial public health problem in the United States. Multiple forms of interpersonal violence, including dating violence, sexual violence, and bullying, negatively affect youths and can continue to have damaging effects throughout a person’s life. The findings in this report are consistent with those in previous studies about disparities in interpersonal violence victimization by demographic characteristics; the report also provides additional insight about the specific groups of students who are at highest risk for particular types of interpersonal violence and who might benefit most from prevention efforts. In addition, the findings increase understanding of the contextual factors associated with interpersonal violence victimization (e.g., frequency, location, and co-occurrence of subtypes) and can guide how violence prevention professionals select and implement prevention approaches for addressing dating violence, sexual violence, and bullying. Prevention approaches at the individual, relationship, and school or community levels (e.g., those that seek to increase youths’ skills in preventing violence, change social norms related to violence, and modify the physical and social environment in schools and communities to increase protection against violence) are crucial for building a comprehensive strategy to reduce interpersonal violence victimization among youths.
